# Expression Profile Analysis to Identify Circular RNA Expression Signatures in Muscle Development of Wu'an Goat Longissimus Dorsi Tissues

**DOI:** 10.3389/fvets.2022.833946

**Published:** 2022-04-18

**Authors:** Zuyang Zhou, Kunyu Li, Jiannan Liu, Hui Zhang, Yekai Fan, Yulin Chen, Haiyin Han, Junqi Yang, Yufang Liu

**Affiliations:** ^1^College of Life Sciences and Food Engineering, Hebei University of Engineering, Handan, China; ^2^School of Landscape and Ecological Engineering, Hebei University of Engineering, Handan, China

**Keywords:** goat, longissimus dorsi, DE circRNA, ceRNA, muscle

## Abstract

The growth and development of skeletal muscle is a physiological process regulated by a variety of genes and signaling pathways. As a posttranscriptional regulatory factor, circRNA plays a certain regulatory role in the development of animal skeletal muscle in the form of a miRNA sponge. However, the role of circRNAs in muscle development and growth in goats is still unclear. In our study, apparent differences in muscle fibers in Wu'an goats of different ages was firstly detected by hematoxylin-eosin (HE) staining, the circRNA expression profiles of *longissimus dorsi* muscles from 1-month-old (mon1) and 9-month-old (mon9) goats were screened by RNA-seq and verified by RT–qPCR. The host genes of differentially expressed (DE) circRNAs were predicted, and Gene Ontology (GO) and Kyoto Encyclopedia of Genes and Genomes analyses (KEGG) of host genes with DE circRNAs were performed to explore the functions of circRNAs. The circRNA-miRNA-mRNA networks were then constructed using Cytoscape software. Ten significantly differentially expressed circRNAs were also verified in the mon1 and mon9 groups by RT–qPCR. Luciferase Reporter Assay was used to verify the binding site between circRNA and its targeted miRNA. The results showed that a total of 686 DE circRNAs were identified between the mon9 and mon1 groups, of which 357 were upregulated and 329 were downregulated. Subsequently, the 467 host genes of DE circRNAs were predicted using Find_circ and CIRI software. The circRNA-miRNA-mRNA network contained 201 circRNAs, 85 miRNAs, and 581 mRNAs; the host mRNAs were associated with “muscle fiber development” and “AMPK signaling pathway” and were enriched in the FoxO signaling pathway. Competing endogenous RNA (ceRNA) network analysis showed that novel_circ_0005314, novel_circ_0005319, novel_circ_0009256, novel_circ_0009845, novel_circ_0005934 and novel_circ_0000134 may play important roles in skeletal muscle growth and development between the mon9 and mon1 groups. Luciferase Reporter Assay confirmed the combination between novel_circ_0005319 and chi-miR-199a-5p, novel_circ_0005934 and chi-miR-450-3p and novel_circ_0000134 and chi-miR-655. Our results provide specific information related to goat muscle development and a reference for the goat circRNA profile.

## Introduction

As crucial agricultural and economic animals in our country, goats provide a large amount of meat for human consumption. Muscle is an important part of the mammalian body, while meat production is closely related to muscle growth and development (1). Skeletal muscle is composed of multinucleated muscle fibers consisting of muscle cells, which account for ~40% of the body weight of mammals (2). The growth and development of skeletal muscle is a physiological process regulated by a variety of transcription factors and signaling pathways, in which the *Pax* gene family (paired-homeobox family), myogenic regulatory factors (*MRFs*) and myocyte enhancer Factor 2 (*MEF2*) interact with each other to regulate the expression of downstream myogenesis-related genes, thereby regulating muscle growth and muscle regeneration (3–5). In addition, the Wnt signaling pathway and the p38/MAPK signaling pathway regulate the expression of myogenic regulatory factors to maintain the balance of proliferation and differentiation during muscle development (6, 7). With the development of whole-transcriptome sequencing technology, an increasing number of noncoding regulatory elements have been brought to the attention of researchers, including miRNAs, lncRNAs, and circRNAs. Studies have shown that noncoding RNAs are involved in cell proliferation, muscle formation and other processes (8, 9). However, there are still large numbers of noncoding RNAs that are worthy of further study.

circRNA is a kind of closed circular noncoding RNA without a 3′ end poly A structure and a 5′ end cap structure whose length can range from hundreds to thousands of bases. CircRNA is not easily degraded by RNA exonuclease and is stable and widely exists in the biological world with evolutionary conservation (10). Although most circRNAs still lack functional annotations, recent research reports suggest that circRNAs may play an important role in gene regulation (11). Studies have shown that circRNAs are widely involved in biological proliferation, regulation of differentiation and signal transduction (12). Broad investigation of the role of circRNA as a posttranscriptional regulatory factor in animal skeletal muscle development and its related regulatory network has begun. Thirty-one significantly different circRNAs were screened out, which laid a foundation for the study of circRNAs in skeletal muscle development (13). Liang et al. identified 149 circRNAs that were potentially associated with postnatal muscle growth using ribosome-depleted RNA-seq (14). In addition, studies in cattle have found that circFGFR4 promotes myoblast differentiation and induces apoptosis, overexpression of circFGFR4 increases the expression of Wnt3a, and miR-107 abolishes this effect. These results indicate that circFGFR4 combined with miR-107 promotes cell differentiation (11). At present, there have been few circRNA studies in goats. Liu et al. found that circ-8073 regulates centrosome protein 55 (CEP55) by sponging miR-449a and promotes the proliferation of goat endometrial epithelial cells through the PI3K/AKT/mTOR pathway (15). Zhang et al. showed that circ-140 was a sponge of chi-miR-8516 and that the circ-140/chi-miR-8516/STC1-MMP1 axis participated in adjusting the production of casein and lipids in goat mammary epithelial cells through the phosphorylation of mTOR and STAT5 (16). There have also been few reports about circRNAs regulating the growth and development of goat skeletal muscle. Therefore, our study has certain significance for exploring the molecular mechanisms of goat muscle development.

A previous study in goats showed that the larger the diameter of skeletal muscle fibers is, the faster the growth rate; the muscle fiber diameter tends to increase between 1 month of age and 9 months of age, while the muscle fiber diameter decreases after 9 months of age (17). However, the mechanisms of goat skeletal muscle development remain unclear. The Wu'an goat, a Chinese native goat breed, is famous for its large size, adaptability, tender meat and light taste (18). In this study, six Wu'an goats between 1 month old and 9 months old were selected to identify circRNAs. High-throughput sequencing technology and functional verification experiments were performed to identify the DE circRNA and its host genes. Then, the target miRNAs of circRNAs were predicted, and a circRNA-miRNA regulatory network and a ceRNA network were constructed. We preliminarily identified the regulatory networks of circRNAs in muscle development. This research provides information that could be used for improving meat yield performance by controlling key factors in regulatory networks.

## Materials and Methods

### Animals and Muscle Tissue Preparation

The Wu'an goats used in this study were all selected from the Hebei Wu'an goat farm. Six nanny goats were grouped into 1-month-old (*n* = 3, mon1 group) and 9-month-old (*n* = 3, mon9 group) groups, and no significant differences in age, weight, or height were noted. The feeding environment and nutrient levels of all goats were the same. Muscle tissue was collected from the *longissimus dorsi* muscle of the goats. All samples were quickly frozen in liquid nitrogen immediately after collection and stored at −80°C until further use.

### HE Slices of Muscle Tissue

Frozen sections were made from muscle tissue fixed with optimal cutting temperature compound (O.C.T.Compound). The HE staining assay included washing with water, hematoxylin staining for 10 minutes, washing with 1 % Hydrochloric acid alcohol, washing and soaking with PBS for 15 min, Soaking with 70% ethanol for 3 minutes, Soaking with 80% ethanol for 3 min, eosin staining for 1minute, washing with 95% ethanol for 1 minute, Soaking with 100% ethanol for 3 minutes, twice, Soaking with xylene and ethanol (1:1) for 5 min, Dewaxing with xylene for 5 minutes, twice, natural drying, sealing, and image acquisition. HE staining images were used for counting, and the diameter was measured by ImageJ (v1.49) software. SPSS software (version 19) was used for statistical analysis to calculate significant differences.

### Library Preparation and RNA Sequencing

High-throughput whole-transcriptome sequencing and subsequent bioinformatics analysis were performed by Novogene (Beijing, China). According to the manufacturer's instructions, total RNA was extracted from the *longissimus dorsi* muscles of 1-month-old (mon1) and 9-month-old (mon9) goats with TRIzol reagent (Tiangen, Beijing, China), and genomic DNA was removed with deoxyribonuclease I (Takara, Dalian, China). Ribosomal RNAs were removed from the total RNA, and the RNAs were then broken into short 250–300-bp fragments. The fragmented RNA was used as a template, random oligonucleotides were used as primers to synthesize the first strand of cDNA, and then the RNA was degraded by RNase H in the DNA polymerase I system. dNTPs (dUTP, dATP, dGTP, and dCTP) were used as raw materials to synthesize the second cDNA strand. The purified double-stranded cDNA underwent end repair, A-tailing, and ligation of a sequencing adapter. AMPure XP beads were used to screen cDNA of ~200 bp, and then the USER enzyme was used to degrade the second strand of cDNA containing uracil. Finally, PCR amplification was carried out, and a library was obtained. Illumina PE150/SE50 (Casava 1.8) sequencing was performed to construct a library.

### Read Quality and Mapping

The default parameters were used to trim and filter the original paired reads to obtain high-quality automatic trimming results. In addition, Qubit 2.0 software was used to align the clean reads with the reference goat genome and to generate the orientation map. The software was used to align ribonucleic acid sequence reads with the genome to detect gene expression and exon splicing. Index of the reference genome was built using STAR and paired-end clean reads were aligned to the reference genome using STAR (v2.5.1b). STAR used the method of Maximal Mappable Prefix(MMP) which can generate a precise mapping result for junction reads.

### Analysis of Differentially Expressed circRNAs

We used TPM (transcripts per million) as a standardized method to quantify the expression of circRNAs, and DEseq2 R package (1.10.1) was used to analyze the differential expression of circRNAs (19). In pairwise comparisons, circRNAs with *P* < 0.05 and an absolute fold change value >1 were considered to be significantly differentially expressed, and finally, the numbers of upregulated and downregulated circRNAs were obtained. The host genes of DE circRNAs were predicted by Find_circ and CIRI software (10, 20).

### Enrichment Analysis by GO, KEGG

Gene Ontology (GO) and KEGG pathway analyses of the host genes of differentially expressed circRNAs were used for annotation. GOseq software was used for GO function analysis. KOBAS software was used to test the statistical enrichment of differential gene expression in KEGG pathways. When *P* < 0.05, GO terms and KEGG pathways were considered to be significantly enriched.

### Prediction of miRNA Targets of circRNAs

To explore the functions of circRNAs and predict which circRNAs act as miRNA sponges, we used miRanda to predict the target relationships. In view of the published reports and the extractability of the sequences, we selected only classical (when the formation site of the circRNA was exactly at the boundaries of exons) and antisense (when the circRNA was formed by the antisense strand of the gene) circRNAs to predict the miRNA targeting relationships (21).

### Network Analysis

Based on the ceRNA theory, we searched for circRNA-gene pairs with the same miRNA binding site and created miRNA-circRNA-mRNA combinations with circRNA (as the decoy), miRNA (as the core), and mRNA (as the target) to construct a ceRNA regulatory network. At the whole-transcriptome level, the ceRNA regulatory network was used to reveal a new mechanism by which ncRNAs regulate gene expression. Interaction diagrams were drawn with Cytoscape software (v3.7.1).

### Experimental Verification of circRNA

Real-time quantitative PCR (RT–qPCR) was used to verify the expression levels of circRNAs. The expression levels of the selected circRNAs were standardized with the levels of the housekeeping gene *RPL19*. Primer 5.0 software was used to design convergent and divergent primers for circRNAs (Table 1), which were synthesized by Beijing Tianyi Huiyuan Biotechnology Co., Ltd. (Beijing, China). Total RNA was converted into cDNA using random hexamers with a Transcriptor First Strand cDNA Synthesis Kit (TAKARA, Japan). RT–qPCR (20 μL) consisted of 2 μL of template cDNA, 0.8 μL of upstream and downstream primers, 10 μL of TB Green Premix Ex TaqII, and 6.4 μL of RNAse-free water. The amplification program was as follows: 94°C for 10 min and 40 cycles of 94°C for 30 s, 60°C for 30 s, and 72°C for 40 s. To determine the existence of circRNAs, the cDNA was collected again after RNase R (Lucigen, USA) digestion according to Liu et al. (21). The expression of circRNAs was calculated using the 2^−ΔΔCT^ value method (22).

**Table 1:** RT–PCR primers for detecting circRNAs.

**Gene Name**	**Primer sequence**	**Length (bp)**	**Tm (**°**C)**
*novel_circ_0007486*	F:5′-GACGAGAACCTGGATGTGGTG-3′	135	60
	R: 5′-TCCCTCTGAGCAGTCTGCTATG-3′		
*novel_circ_0009845*	F:5′-GGAAAAAGAGCATGAAGCTTTG−3′	149	60
	R:5′-AGCTGGGGTCACAGTCAATAAG-3′		
*novel_circ_0008755*	F:5′-TTAGATGTACGGTCTCAGCGG-3′	113	60
	R:5′-GGAGATTCACCTCCATTCAGG-3′		
*novel_circ_0008479*	F:5′-TGGTTGGTGTTTTGGGTTATG-3′	113	60
	R:5′-TAGCAAATACATCCCCAGCAT-3′		
*novel_circ_0002141*	F:5′-CGTTTGAGTTTGGCTTCACATT-3′	225	60
	R:5′-TCAGGATTTTTATCTGGATGCC-3′		
*novel_circ_0005934*	F:5′-GAAACTGGGAGCCTAGTTATGG-3′	165	60
	R:5′-TTACAGTGTTGGGAACGCTTG-3′		
*novel_circ_0005314*	F:5′-GGACAGATGATGAGAGAGCACC-3′	127	60
	R:5′-TGGAAGACATGAGCTCGAGTG-3′		
*novel_circ_0003615*	F:5′-TCTCAGATGAAGGCATGATCG-3′	157	60
	R:5′-TCTAAACAAAGGTCACGCCAG-3′		
*novel_circ_0002779*	F:5′-CTGAGGAAGCGGCTAACACC-3′	233	60
	R:5′-CTGGCTGGCTCTTTGTCACC-3′		
*novel_circ_0002766*	F:5′-TGGACAAGGCTTTGCATTAGTT-3′	187	60
	R:5′-AGTAGAGCAGATTTTCCAACGC-3′		
*RPL-19*	F:5′-ATCGCCAATGCCAACTC-3′	154	60
	R:5′-CCTTTCGCTTACCTATACC-3′		

### Luciferase Reporter Assay

The miRNA binding site of novel_circ_0005319, novel_circ_0005934 and novel_circ_0000134 were screened according to the analysis results obtained with ENCORI software. Cells were seeded in 24-well plates at a density of 1 × 10^4^ cells (HEK-293T) per well 24 h before transfection. Then, the cells were transfected with a mixture of 200 ng of circRNA-WT target or circRNA-MUT target, and 10 μL of miRNA mimic or mimic-NC was utilized with Lipofectamine 2000 (Invitrogen, USA). In this study, the double luciferase vector pmir-GLO was selected (Genewiz, Suzhou, China), and the novel_circ_0005319, novel_circ_0005934 and novel_circ_0000134 sequence were cloned into the reporter gene vector to synthesize the predicted miRNA mimics and control. The miRNA mimic was obtained from RiboBio (Guangzhou, China). After 48 h, luciferase activity was measured with a dual luciferase reporter assay system (Promega, Madison, WI, United States). The expression levels of reporter genes were detected by a multifunctional enzyme labeling instrument, and the assays were performed in triplicate.

### Statistics

The RT–qPCR data were analyzed using SPSS software version 19. A paired *t*-test and two-way analysis of variance were performed to analyze the relative expression levels of genes. A *P*-value < 0.05 was considered a significant difference.

## Results

### Apparent Differences in Muscle Fibers in Wu'an Goats of Different Ages

**Figure 1:**
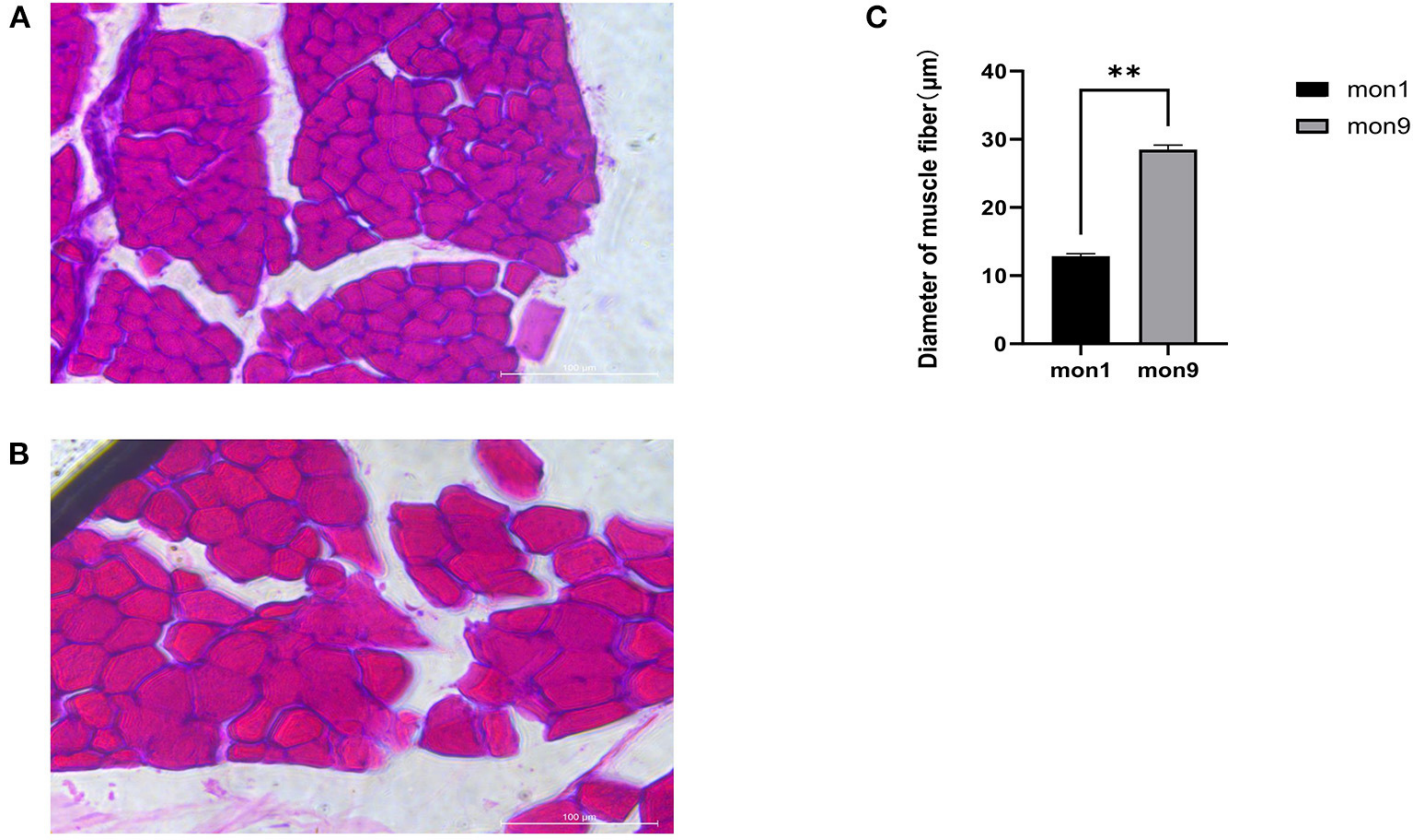
HE staining of muscle tissue frozen sections. **(A)** muscle fibers of mon1 group. **(B)** muscle fibers of mon9 group. **(C)** diameter comparison of muscle fibers.

The muscle fibers of the *longissimus dorsi* in the Wu'an goats at different ages were significantly different according to the observation of frozen sections stained by HE (Figures 1A,B). The diameter of single muscle fibers of the mon9 group was significantly wider than that of the mon1 group, and the shape of each muscle fiber was also greater. ImageJ software analysis showed that there were significant differences in the muscle fiber diameter (*P* < 0.01) (Figure 1C).

### Summary of circRNA Profiles From Goat Longissimus Dorsi Muscle

To examine the expression profiles of circRNAs in the *longissimus dorsi* muscle of Wu'an goats at different ages, we established a cDNA library of six *longissimus dorsi* muscle samples from goats at the ages of 1-month-old (*n* = 3, mon1 group) and 9-month-old (*n* = 3, mon9 group) groups. We obtained the raw data after Illumina sequencing. After removing the low-quality reads and the reads with adapter sequences from the RNA-seq raw reads, 583,214,544 clean reads (mon1: 296,548,474, mon9: 286,666,070) were obtained (Table 2). In the mon1 and mon9 samples, 94.8 and 92.48% of the reads were located in the reference genome, and the clean (Q30) base rates were 92.50 and 92.56%, respectively.

**Table 2:** Read quality for RNA-seq.

**Sample name**	**RAW Reads**	**CLEAN Reads**	**Raw Bases (G)**	**Clean Bases (G)**	**Error rate (%)**	**Q20 (%)**	**Q30 (%)**	**GC Content (%)**
mon1_1	98,721,676	9,703,6650	14.81	14.56	0.03	97.47	93	52.99
mon1_2	104,194,360	101,986,738	15.63	15.3	0.03	97.29	92.64	55.08
mon1_3	99,110,136	97,525,086	14.87	14.63	0.03	96.95	91.87	53.06
mon9_1	98,408,274	96,645,866	14.76	14.5	0.03	97.4	92.79	51.64
mon9_2	90,534,786	8,8937,344	13.58	13.34	0.03	97.48	92.97	53.35
mon9_3	10,615,6574	10,108,2860	15.92	15.16	0.03	96.92	91.93	54.31

### Identification of Differentially Expressed circRNAs

**Figure 2:**
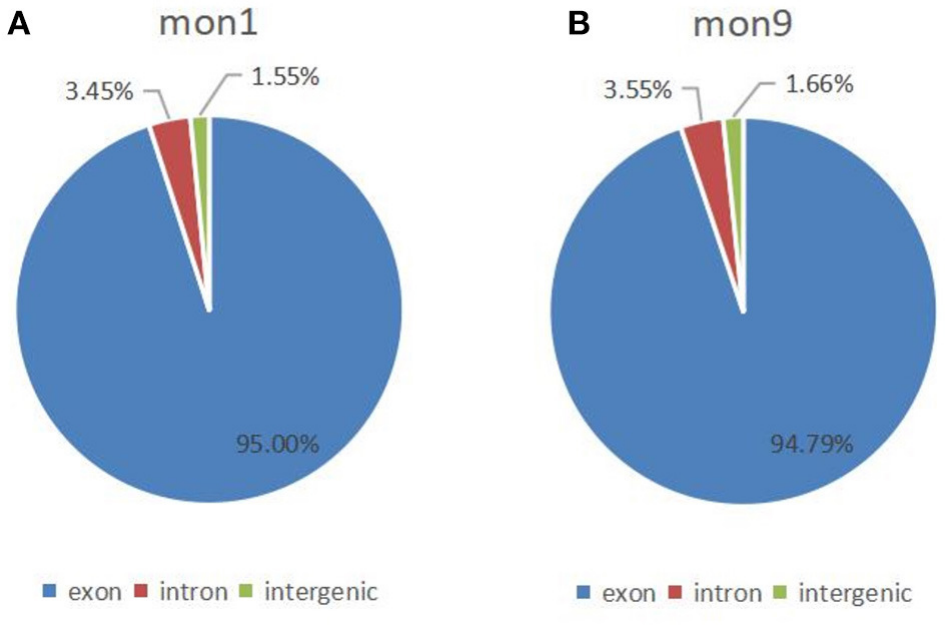
Distribution of circRNAs in the genome. **(A)** Distribution of circRNAs at 1 month of age; **(B)** Distribution of circRNAs at 9 months of age.

**Figure 3:**
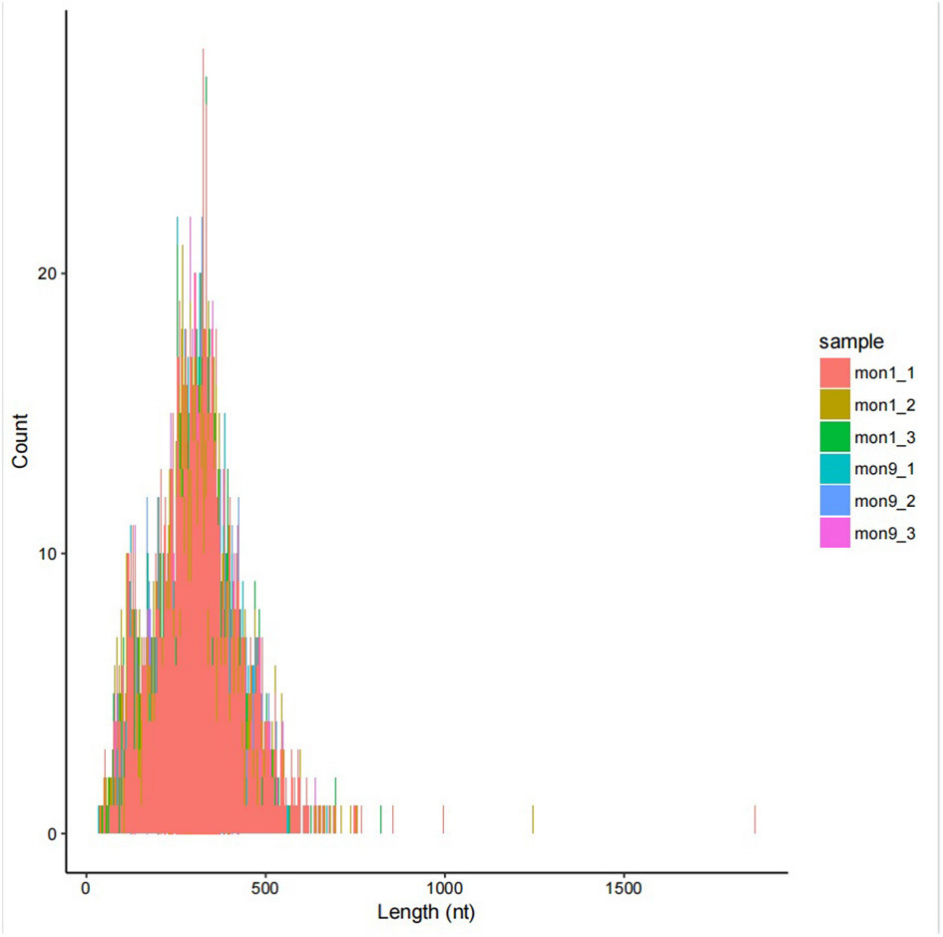
Circos diagram of the density distribution of all circRNAs on the chromosomes of each sample.

**Figure 4:**
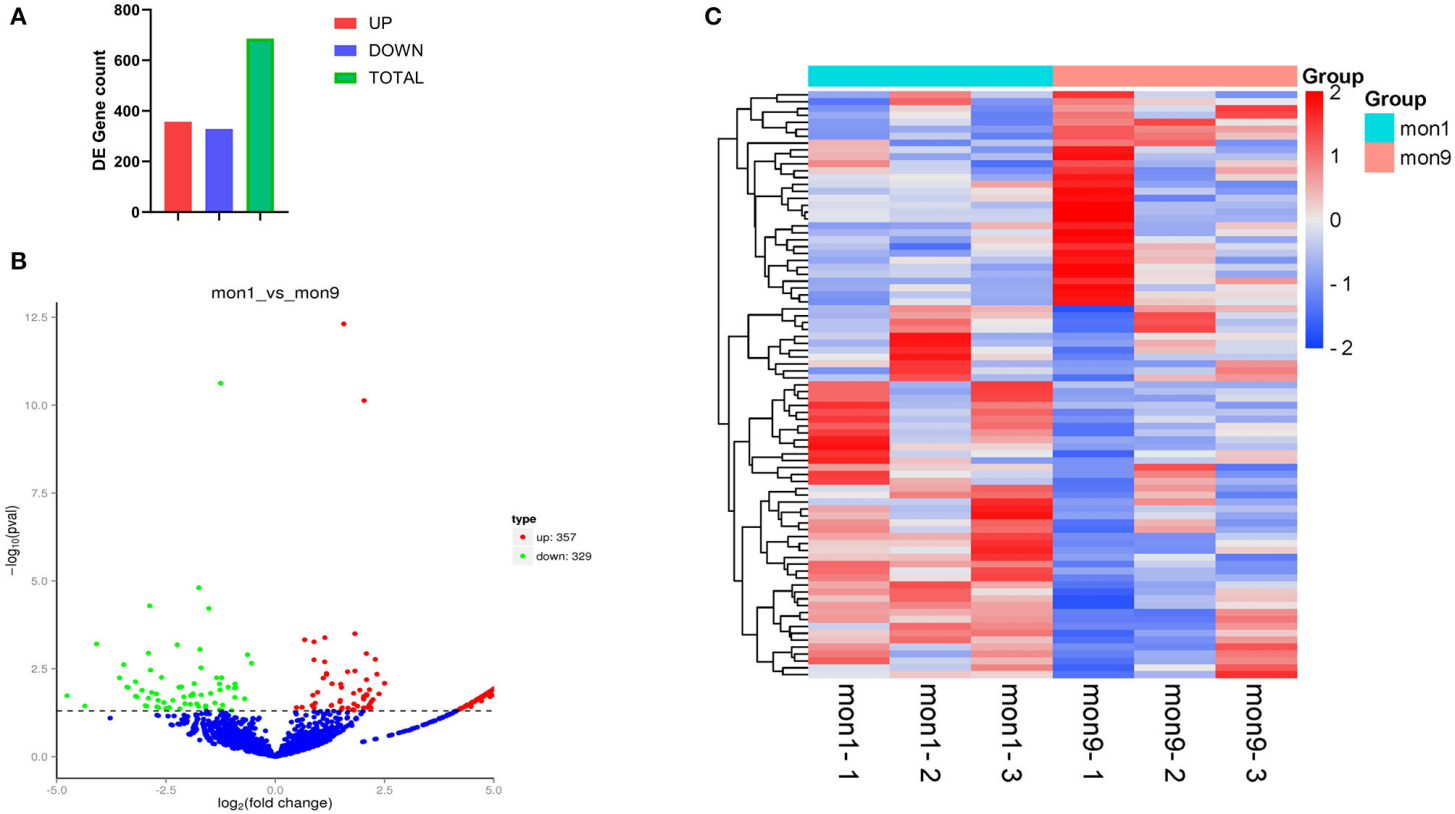
Differentially expressed circRNAs. **(A)** All 686 differentially expressed circRNAs, of which 357 were upregulated and 329 were downregulated. **(B)** Visualization of the differential expression in a volcano graph. The abscissa is the fold change of expression in the mon1 and mon9 samples, and the ordinate is the statistical significance of the expression change. Different colors indicate different classifications: green (downregulated) and red (upregulated). **(C)** Clustering heatmap of differentially expressed genes. The change in expression level is indicated by the change in color, with blue indicating a lower expression level and red indicating a higher expression level.

In this study, 6,075 circRNAs and 2,893 parental genes were detected, of which 1,207 (19.87%) were expressed in all samples (Supplementary Table 1). According to the positions of circRNAs in the genome, they were divided into exons, introns, and intergenic circRNAs. Most (94.90%) circRNAs were derived from exons, a few (3.50%) were derived from introns, and very few were derived from intergenic regions (1.6%) (Figure 2). The lengths of the circRNAs identified in this study ranged from 35 to 1,866 bp (Supplementary Table 2), and most (94.97%) circRNAs were less than 500 bp in length (Figure 3). In this study, 686 differentially expressed circRNAs and 467 host genes (Supplementary Table 3) were detected, of which 357 circRNAs were upregulated and 329 were downregulated (Figure 4A). A red volcano map (Figure 4B) and a cluster heatmap (Figure 4C) were generated to visualize the distribution of the differentially expressed circRNAs.

### GO Enrichment and KEGG Pathway Analyses

**Figure 5:**
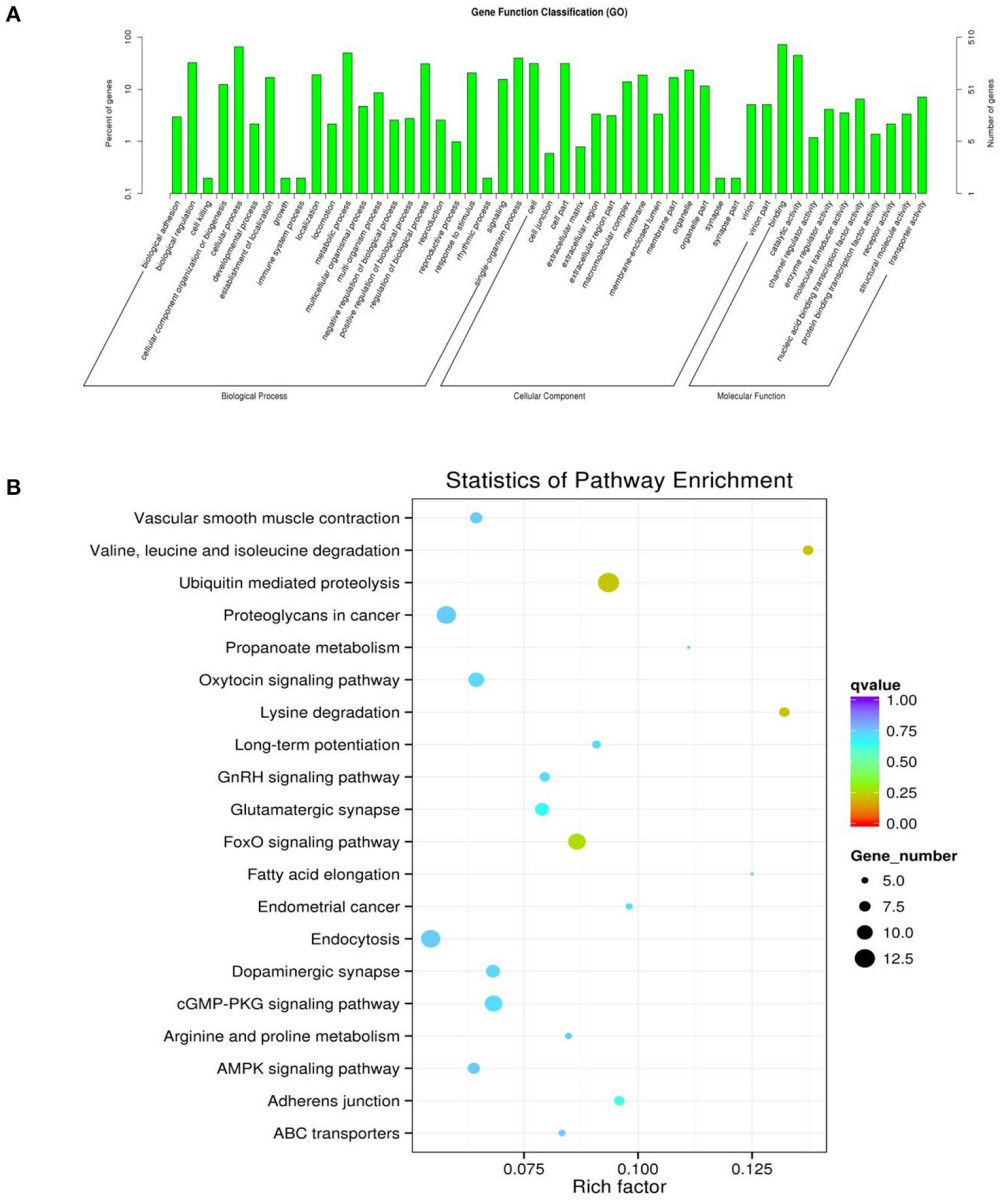
GO and KEGG diagrams. **(A)** Forty-nine different GO term annotations of the parental genes of differentially expressed circRNAs. The abscissa is the ontology classification, and the ordinate is the proportion of genes annotated to this term among all the annotated genes. **(B)** The parental genes of the differential circRNAs for the 20 pathways that are significantly enriched; the closer the color is to red, the higher the enrichment degree. The size of each dot indicates the number of genes enriched in the KEGG entry. The larger the dot is, the more genes enriched in the KEGG entry, and vice versa.

To determine the functions of these genes, GO analysis was performed. Since there is currently no annotation information for the novel circRNAs identified in our study, we annotated the host genes of the circRNAs instead of the circRNAs according to the statistical data of differentially expressed circRNAs and their host genes. The host genes of the differentially expressed circRNAs were annotated with 49 different GO terms (Figure 5A). The highest enrichment scores were cellular process (GO:0009987), metabolic process (GO:0008152), single-organism process (GO:0044699), cell (GO:0005623), cell part (GO:0044464), binding (GO:0005488), and catalytic activity (GO:0003824). To predict the functions of the significantly enriched host genes, pathway analysis was performed based on the KEGG pathway database. Among the 20 pathways that were significantly enriched, valine, leucine and isoleucine degradation, ubiquitin-mediated proteolysis, ubiquitin-mediated proteolysis, proteoglycans in cancer, oxytocin signaling pathway, endocytosis, FoxO signaling pathway and GMP-PKG signaling pathway were most significantly enriched (Figure 5B).

### Confirmation of circRNA Expression by RT–qPCR

**Figure 6:**
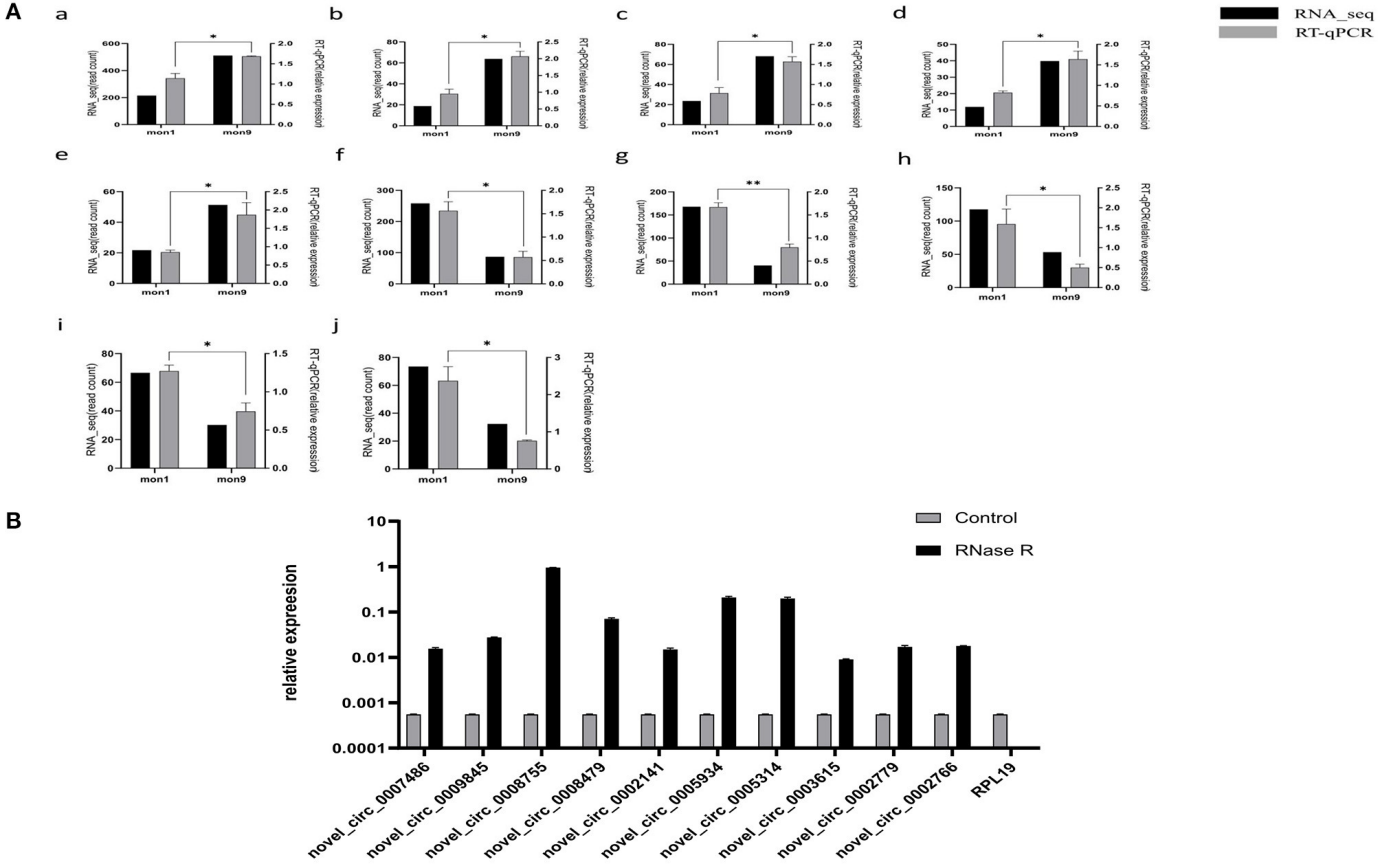
Expression verification of circRNAs. **(A)** RT–qPCR verification of mon1 and mon9 differentially expressed circRNAs in RNA-seq. Graph numbers of a, b, c, d, e, f, g, h, i and j represent novel_circ_0007486, novel_circ_0009845, novel_circ_0008755, novel_circ_0008479, novel_circ_0002141, novel_circ_0005934, novel_circ_0005314, novel_circ_0003615, novel_circ_0002779 and novel_circ_0002766, respectively. **(B)** The expression level of circRNAs and *RPL19* After RNase R digestion.

To verify the accuracy of the RNA-seq data, we randomly selected 10 differentially expressed circRNAs with log2 |fold change| > 1 and *P* < 0.01. The differentially expressed circRNAs, including five upregulated circRNAs (novel_circ_0005934, novel_circ_0005314, novel_circ_0003615, novel_circ_0002779, novel_circ_0002766) and five downregulated circRNAs (novel_circ_0007486, novel_circ_0009845, novel_circ_0008755, novel_circ_0008479, novel_circ_0002141), were selected for RT–qPCR analysis. The results showed that the RT–qPCR results were consistent with the expression trends observed in the RNA-seq data (Figure 6A), indicating that the RNA-seq results were reliable. After RNase R digestion, the presence of circRNA was still detected by qRT-PCR, while as a linear RNA, the expression of the control group (*RPL19*) was almost undetectable after digestion (Figure 6B). The results indicated that the circRNAs we detected were circular RNAs.

### Construction of a ceRNA Network of Goat Muscle DE circRNAs

**Figure 7:**
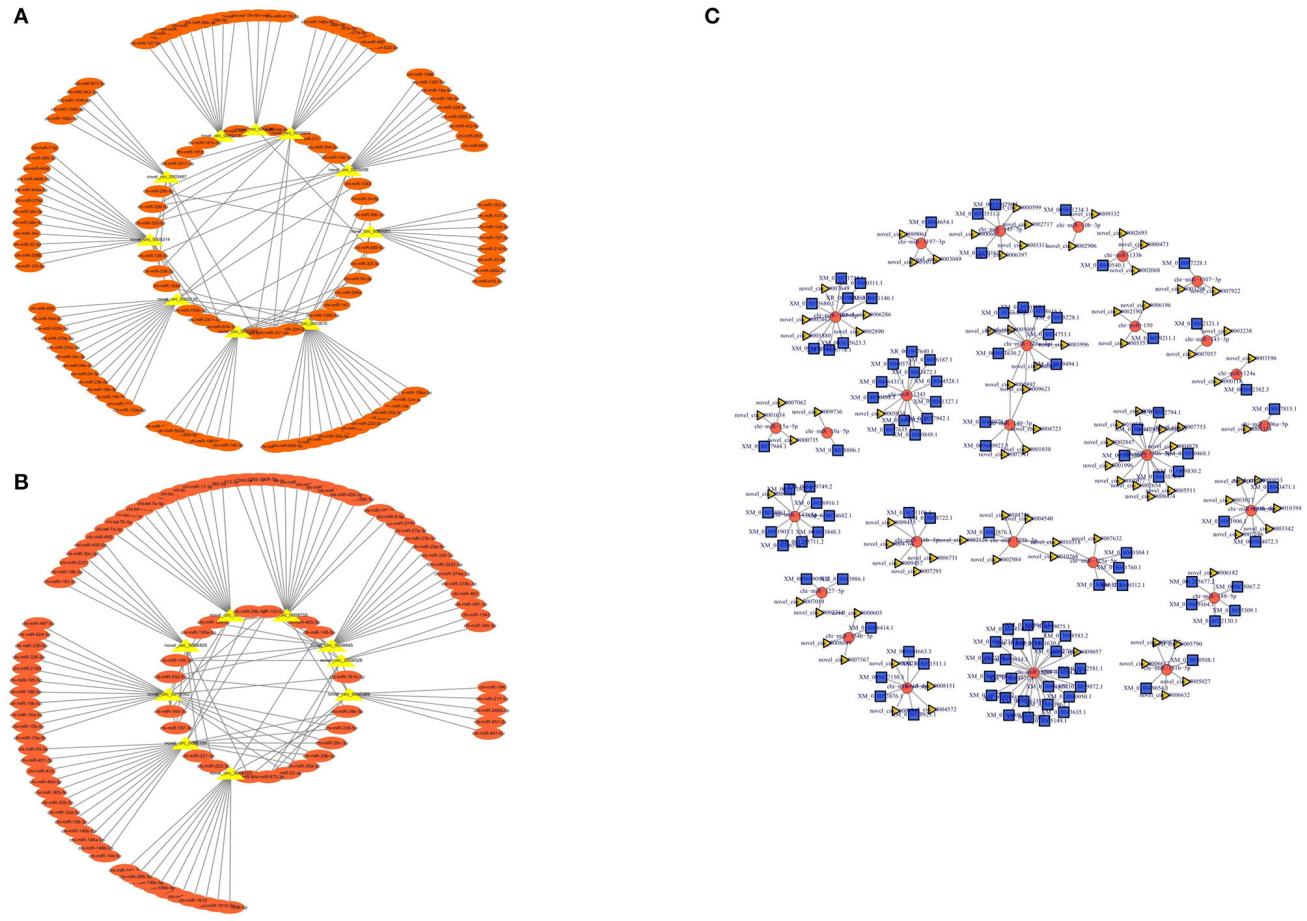
circRNA-miRNA interaction network. **(A)** Significantly upregulated circRNAs and their targeted miRNAs. **(B)** Significantly downregulated circRNAs and their targeted miRNAs. **(C)** The circular RNA-miRNA-mRNA interaction network between circular RNAs and their target genes.

To further understand the functions of differentially expressed circRNAs, we used miRanda to predict the interactions between circRNAs and miRNAs. The targeted miRNAs of ten significantly upregulated circRNAs are shown in Figure 7A, while Figure 7B shows the targeted miRNAs of nine significantly downregulated circRNAs. Then, a competing endogenous RNA (ceRNA) network was constructed based on circRNA-miRNA interactions and miRNA-mRNA interactions, which contained 201 circRNAs, 85 miRNAs, and 581 mRNAs (Supplementary Table 4). The top 200 interaction networks were selected for construction, and the ceRNA network was visualized using Cytoscape software (Figure 7C). This network might provide a comprehensive perspective of the interactions between miRNAs, circRNAs and mRNAs in the development of muscle fibers in goats of different ages.

### Detection of Luciferase Activity

**Figure 8:**
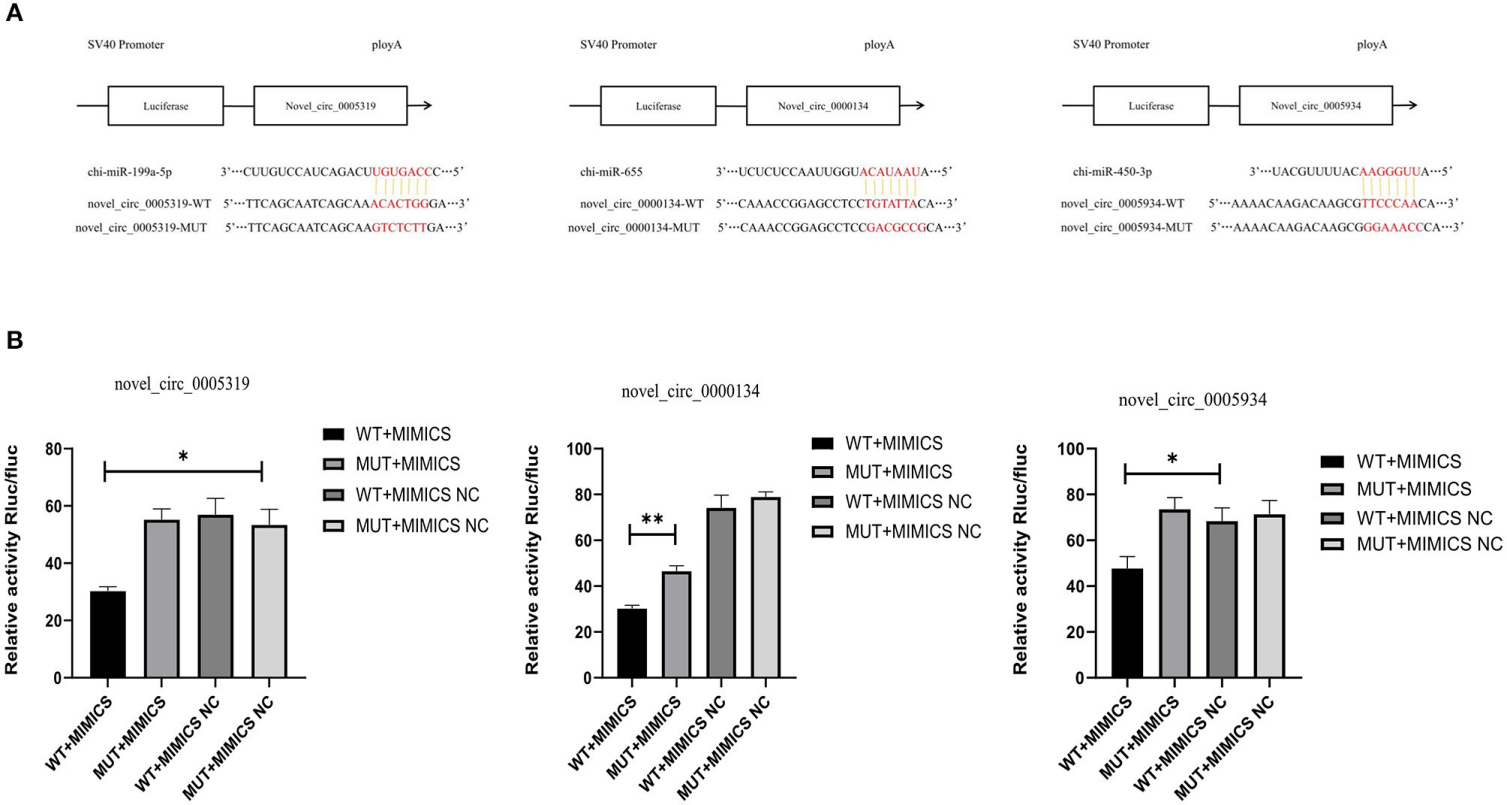
The prediction and verification binding sites of the circRNAs. **(A)** The predicted binding sites of miRNA-circRNA pairs. **(B)** Verification of dual Luciferase reporter assay.

Novel_circ_0005319 contained a chi-miR-199a-5p binding site, novel_circ_0005934 contained a chi-miR-450-3p binding site and novel_circ_0000134 contained a chi-miR-655 binding site (Figure 8A). The results of the double luciferase reporter gene system showed that the luciferase activity of chi-miR-199a-5p and pmirGLO-novel_circ_0005319,chi-miR-450-3p and pmirGLO-novel_circ_0005934, chi-miR-655 and pmirGLO-novel_circ_0000134 were significantly lower than that of the control groups (*P* < 0.05), but the luciferase activity of the mutant vector and novel_circ_0005319, novel_circ_0005934, novel_circ_0000134 cotransfected groups were not significantly different from that of the control group (Figure 8B), indicating that novel_circ_0005319 has a chi-miR-199a-5p binding site, novel_circ_0005934 contained a chi-miR-450-3p binding site and novel_circ_0000134 contained a chi-miR-655 binding site.

## Discussion

With the development of high-throughput technology, circRNA has been identified as a new type of noncoding RNA (23). Although thousands of unique circRNAs have been identified in cell types of different species, especially in humans and mice (24–28), in which they have been shown to play important roles in animal development and growth, circRNAs in goats are still not sufficiently elucidated.

Current research on the function of circRNAs is mainly focused on competitive endogenous RNAs. CircRNA can be used as a sponge to adsorb miRNA, thereby affecting posttranscriptional regulation (29–31). In addition, studies have found that circular RNA (circHIPK3) regulates cell growth through sponge adsorption of 9 miRNAs with 18 potential binding sites (12). In this study, we found that some circRNAs also contain multiple target sites of different miRNAs. For example, novel_circ_0009405 contained the targets of chi-miR-143-3p, chi-miR-143-3p, chi-miR-18b-3p, chi-miR-18b-3p, chi-miR-197-3p, chi-miR-221-3p, chi-miR-222-3p, chi-miR-2331, chi-miR-30c-3p, chi-miR-455-5p, chi-miR-455-5p, chi-miR-532-3p and chi-miR-660. In our research, we identified a set of circRNA-miRNA pairs in which chi-miR-127-5p (target of novel_circ_0005319), chi-miR-199a-5p (target of novel_circ_0008103 and novel_circ_0005319), chi-miR-665 (target of novel_circ_0009256), chi-miR-22-5p (target of novel_circ_0005314) and chi-miR-433 (target of novel_circ_0005186) might play a role in the development of muscle fibers. The expression of miR-127-5p in human muscle has been shown to be negatively correlated with the translation efficiency of the β-F1-ATPase protein (32). Another study found that miR-127-5p directly targeted *NAMPT*, which promoted the viability of osteoarthritis chondrocytes and inhibited cell apoptosis, inflammation and extracellular matrix degradation (33, 34). Jia et al. researched and screened the differentially expressed miRNAs in skeletal muscle tissue of sika deer at 1, 3, 5 and 10 years old (35). Among them, miR-199a-5p might play a key role in muscle aging. In addition, some researchers have observed that the overexpression of miR-199a-5p can inhibit the proliferation and migration of vascular smooth muscle cells, which can also suppress their apoptosis (36). Compared with traditional small-tailed Han sheep, Qianhua Mutton Merino is a new breed of sheep for which meat performance has been improved. In research on the Transcriptome Atlas of *longissimus dorsi* of Qianhua Mutton Merino and Small-Tail Han sheep, Sun et al. identified the differentially expressed oar-miR-655-3p and showed that it may play vital roles in muscle growth and development (37). Dickkopf-1 (*DKK1*) is a powerful typical antagonist of the Wnt signaling pathway, and its expression is highly correlated with bone mass and osteoblast maturation. Tang et al. found that miR-433-3p reduces *DKK1* expression and induces osteogenic cell differentiation (38). In addition, Chao et al. sequenced the *longissimus dorsi* muscles of sheep from the 60, 90, and 120th days of pregnancy and the 0 and 360th days after birth and identified the differentially expressed miR-433. These findings suggest that miR-433 might affect sheep skeletal muscle development by regulating myogenic differentiation (39). Qin et al. found that the overexpression of miR-22-5p in rat cardiomyocytes can affect the RAP1/ERK pathway and cardiomyocyte apoptosis (40). Therefore, as potential ceRNAs, circRNAs (novel_circ_0005319, novel_circ_0009256, novel_circ_0005314, novel_circ_0008103, novel_circ_0005186) that can interact with a variety of miRNAs may play a broad endogenous regulatory role in muscle growth and development.

In this study, the GO terms were mainly enriched in biological processes, such as cellular processes, metabolic processes, and the regulation of biological processes. This pattern of enrichment suggested that DE circRNAs may be closely related to the growth and development of skeletal muscle cells. Based on the KEGG pathway database, we further analyzed circRNAs, and we identified 5 different enrichment pathways related to muscle growth and development, including the degradation of valine, leucine and isoleucine, ubiquitin-mediated proteolysis, glutamatergic synapses, which were related to muscle cell differentiation and proliferation (41), the AMPK signaling pathway and the FoxO signaling pathway, which affected muscle fiber processes (42, 43).

CircRNAs play an important role by regulating the transcription and expression of their host genes (44). According to statistical data, we also found several significantly different host genes of circRNAs, including *HOMER1* (host gene of novel_circ_0005314 and novel_circ_0005319), *CDYL* (host gene of novel_circ_0009256), *INPP5F* (host gene of novel_circ_0009845), *LMO7* (host gene of novel_circ_0005934), and *PPP2R3A* (host gene of novel_circ_0000134), which might be involved in regulating the growth and development of muscle cells. Li et al. analyzed the differential expression of the circRNA regulatory network in the skeletal muscles of Large White pigs and Mashen pigs at different ages and confirmed that circRNAs derived from Homer scaffold protein 1 (HOMER1) exons were differentially expressed during skeletal muscle growth, which might have certain significance for increasing the growth rate of pigs and improving meat quality (45). In a mouse knockout (KO) model, the excision of *Homer1* resulted in obvious skeletal muscle myopathy, while in a study of rats, Jia et al. found that when the expression of *Homer1* was downregulated, it inhibited the migration and proliferation of vascular smooth muscle cells (VSMCs) (46, 47). Zhang et al. studied the effect of circRNA *CDYL* on myocardial angiogenesis after acute myocardial infarction and found that overexpression and downregulation of circRNA *CDYL* can promote and inhibit the proliferation of cardiomyocytes cultured *in vitro* (48). INPP5F is called inositol phosphatase 2 and contains the Sac domain, which is essential for the activity of inositol polyphosphate phosphatase (49). Zhu et al. found that in mice, *INPP5F* was an important endogenous regulator of cardiomyocyte size and cardiac stress response (50). Zhou et al. found that the HincII polymorphism of the pig *INPP5F* gene was associated with average daily gain (ADG) from birth to market (150 days) and ADG of 30–100 kg (51). These studies indicate that *INPP5F* is involved in cell proliferation and differentiation. *LMO7* is a multifunctional protein found in the nucleus, cytoplasm and adhesion junctions of many tissues and is expressed at high levels in skeletal muscle and heart (52, 53). Some researchers have found that the gene *LMO7* is related to muscle development in studies of the genetic determinants of Duroc pig carcass traits (54), which are related to muscle development, and its deletion in mice can lead to muscle degeneration and growth retardation (55). In addition, in the absence of *LMO7*, chicken myogenesis was impaired, myotubes decreased and the number of monocytes decreased (56). Protein phosphatase 2 regulatory subunit B″α (*PPP2R3A*) has been found to regulate the Wnt signaling cascade and 5′adenosine monophosphate-activated protein kinase (AMPK) activity, which have functions such as enhancing cell proliferation and migration (57, 58). Some researchers have performed transcriptome sequencing on the *longissimus dorsi* muscles of different breeds and identified the differentially expressed circRNA host gene *PPP2R3A* (21), and Chen et al. found that knocking out *PPP2R3A* could significantly inhibit the proliferation of liver cancer cells, while the overexpression of *PPP2R3A* promoted the proliferation of two liver cancer cell lines (59). The source genes of these circRNAs (novel_circ_0005314, novel_circ_0005319, novel_circ_0009256, novel_circ_0009845, novel_circ_0005934 and novel_circ_0000134) might be involved in regulating the growth and development of muscle cells, whose specific functions require further investigation.

## Conclusion

In summary, differential circRNA expression profiles in goats of different ages were established, and a total of 686 differentially expressed circRNAs were screened. We also screened out some circRNAs that may be involved in the regulation of muscle growth and development as well as their targeted miRNAs and host genes, such as novel_circ_0005319, novel_circ_0008103, novel_circ_0009256, novel_circ_0005314, novel_circ_0005186, novel_circ_0009845, novel_circ_0005934 and novel_circ_0000134. Our research will aid in understanding the function of circRNAs in goats and may provide clues for the study of goat muscle development.

## Data Availability Statement

The datasets presented in this study can be found in online repositories. The names of the repository/repositories and accession number(s) can be found below: https://www.ncbi.nlm.nih.gov/, PRJNA749569.

## Ethics Statement

The animal study was reviewed and approved by Institutional Animal Care and Use Committee at Hebei University of Engineering (AEEI-16015).

## Author Contributions

ZZ and YL: conceptualization, formal analysis, data curation, and writing-original draft preparation. YL, ZZ, and KL: methodology. ZZ: validation. HZ, YF, ZZ, YC, and YL: investigation. JL, HH, JY, HZ, and YL: resources. YL: supervision. HH, JY, and YL: project administration. JY and YL: funding acquisition. All authors have read and agreed to the published version of the manuscript.

## Funding

This research was funded by the National Natural Science Foundation of China Youth Program (32102509) and Natural Science Foundation of Hebei Province of China for Youths (C2019402261 and C2019402293).

## Conflict of Interest

The authors declare that the research was conducted in the absence of any commercial or financial relationships that could be construed as a potential conflict of interest.

## Publisher's Note

All claims expressed in this article are solely those of the authors and do not necessarily represent those of their affiliated organizations, or those of the publisher, the editors and the reviewers. Any product that may be evaluated in this article, or claim that may be made by its manufacturer, is not guaranteed or endorsed by the publisher.

## Supplementary Material

Supplementary Table 1 All circRNAs and their host genes identified in this study.

Supplementary Table 2 Length data of all identified circRNAs.

Supplementary Table 3 Results of differentially expressed circRNAs.

Supplementary Table 4 Correlation analysis results of miRNAs and circRNAs that have a targeting relationship with miRNAs and mRNAs that have a targeting relationship with miRNAs.
